# Development of Sound Localization Strategies in Children with Bilateral Cochlear Implants

**DOI:** 10.1371/journal.pone.0135790

**Published:** 2015-08-19

**Authors:** Yi Zheng, Shelly P. Godar, Ruth Y. Litovsky

**Affiliations:** Waisman Center, University of Wisconsin Madison, Madison, Wisconsin, United States of America; Birkbeck College, UNITED KINGDOM

## Abstract

Localizing sounds in our environment is one of the fundamental perceptual abilities that enable humans to communicate, and to remain safe. Because the acoustic cues necessary for computing source locations consist of differences between the two ears in signal intensity and arrival time, sound localization is fairly poor when a single ear is available. In adults who become deaf and are fitted with cochlear implants (CIs) sound localization is known to improve when bilateral CIs (BiCIs) are used compared to when a single CI is used. The aim of the present study was to investigate the emergence of spatial hearing sensitivity in children who use BiCIs, with a particular focus on the development of behavioral localization patterns when stimuli are presented in free-field horizontal acoustic space. A new analysis was implemented to quantify patterns observed in children for mapping acoustic space to a spatially relevant perceptual representation. Children with normal hearing were found to distribute their responses in a manner that demonstrated high spatial sensitivity. In contrast, children with BiCIs tended to classify sound source locations to the left and right; with increased bilateral hearing experience, they developed a perceptual map of space that was better aligned with the acoustic space. The results indicate experience-dependent refinement of spatial hearing skills in children with CIs. Localization strategies appear to undergo transitions from sound source categorization strategies to more fine-grained location identification strategies. This may provide evidence for neural plasticity, with implications for training of spatial hearing ability in CI users.

## Introduction

The number of children receiving cochlear implants (CIs) in both ears (bilateral CIs; BiCIs) has grown in recent years. This clinical trend is, in part, motivated by the fact that post-lingually deafened adults demonstrate significant benefits on measures of sound localization, and speech understanding in the presence of interfering stimuli, when using BiCIs compared with a single-CI listening mode [1–3]. Studies on spatial hearing in children who are fitted with BiCIs offer opportunities to understand both basic neuroscience issues, and clinically relevant issues. Early research in this population showed that, during the first year after bilateral activation, performance on spatial hearing tasks is better with BiCIs than with a single CI [4]. At a clinical level, parents on behalf of their children are making decisions as to whether the children should receive one or two CIs. What remains to be better understood is the extent to which children with BiCIs will be able to function similarly to children with normal hearing (NH).

The bilaterally implanted pediatric population offers a compelling opportunity to investigate whether spatial hearing skills can emerge in a human whose auditory system was deprived of hearing at birth. More so, many of the children received sequential implantation, thus, they experienced a period of monaural deprivation during the time that the first CI was activated; subsequently they transitioned to hearing bilaterally. The unique experiences of these children who are fitted with BiCIs may influence the development of spatial hearing skills and binaural encoding capacity. There is evidence that humans [5] and animals [6, 7]learn to localize sound early during development, and that experience-induced plasticity is greatest during this time period. These studies indicate that neural circuitry underlying sound localization is calibrated through ongoing experience with available spatial cues.

In humans, behavioral studies of sound localization typically use two different psychophysical methods. One type of task measures the smallest sound source separation in space that subjects can reliably discriminate. This type of a “relative” task is exemplified by the measure of minimum audible angle (MAA) [8]. Indeed, it has been found that children who received BiCIs after age 4 exhibited MAA thresholds near 20° [9]; in contrast, best-performing NH children have MAA thresholds as low as 3° by 2 years of age [9] and 1-2° by 5 years of age[10]. The average thresholds are approximately 6° for stimuli presented at static levels [9]. In 2-year old BiCIs users whose CIs were activated by age 12 months, MAAs can be within normal limits, but this is not the case for all children [4]. Although the MAA task can be used to evaluate subjects’ spatial acuity, it provides little information regarding subjects’ ability to map spatial locations, nor regarding localization accuracy.

Absolute sound localization is a measure of listeners’ ability to specify the absolute location of a sound source. Several studies with children who use CIs have implemented this approach to investigate spatial hearing skills in the horizontal plane. A standard metric for quantifying listeners’ performance is the root mean square (RMS) error over the entire source array, with lower RMS values representing better performance. Litovsky et al. [2] were the first to implement this approach in pediatric BiCIs users, testing the first 3 children in this unique population using an array of 15-loudspeakers, spanning from -70° to 70°. These children were 8–12 years old at the time that they received the second CI, and only had 3 months of experience with the second CI. Results showed mean RMS error near 55°, suggesting that their spatial hearing skills were poorly developed. A larger number of bilaterally implanted children were tested by Grieco-Calub and Litovsky [11] (N = 21, ages 5–14 years) and Van Deun et al. [12] (N = 30, ages 4–15 years). Grieco-Calub and Litovsky used a single spondaic speech stimulus and Van Deun et al. used a broadband (bell-ring) stimulus. RMS errors fell into similar ranges in these two studies: 19–56° [11] and 13–63° [12].

**Fig 1:**
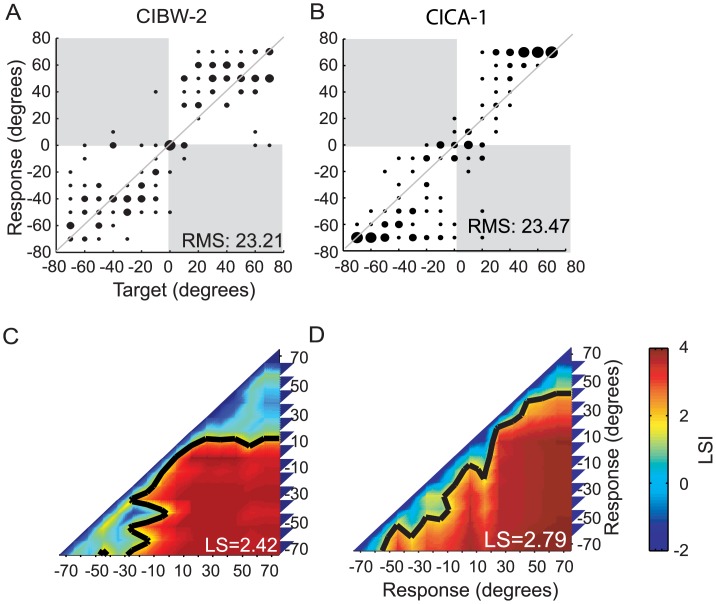
Two examples from the BiCIs group show different response patterns with nearly identical RMS error. Panels A-B display scatter plots for sound source identification with the 15-loudspeaker array. The diameter of the dots is proportional to the frequency of the responses for a given target source. Data falling on the diagonal line represent perfect performance. The numbers inside each plot indicate the overall RMS error. Panels C-D display examples of spatial mapping representation from A-B. Each row and column of the matrix represents the localization sensitivity index (LSI) between one of the 15 response groups to all the 15 response groups (Responses to a given sound source target are considered as a response group). Colors denote LSI. Red shades indicate high LSI, where blue shades indicate low LSI. The matrix is symmetrical about the diagonal line with the element equal to the index between two response groups. The diagonal elements (LSI between itself) are always negative infinite. The black contours indicate threshold sensitivity (LSI = 1.65). The numbers inside each plot indicate the overall averaged localization sensitivity (LS).

One limitation of using RMS error as a single metric over the entire loudspeaker array is that the same value can potentially be obtained for response profiles that demonstrate different types of errors and variability[11, 13]. In addition, RMS error as a single metric of performance, averaged over the entire stimulus space, reflects only partial information about performance, thus the RMS metric is not sufficiently informative regarding details of spatial contiguity among the sound sources, and is not informative about how subjects map acoustic space to spatially-relevant perceptual representations. In Fig 1, we show two examples that illustrate this point. Although the two examples show very different response patterns, the resulting RMS errors are nearly identical. In the example in Fig 1A, most responses are roughly uniformly distributed around ±40°. In contrast, for the example in Fig 1B, few of the responses are near the actual stimulus location and when errors occur they are large, and biased to the extremely distal edges (±70°). It is clear that different response profiles and distributions can result in similar RMS values.

To our knowledge, there is no established quantitative metric for examining the spatial mapping representation of acoustic space. Accurate quantification of spatial mapping representation is important for understanding the auditory mechanisms involved in spatial hearing. This is of particular interest when considering a population of children who underwent a period of auditory deprivation early in life, and experienced onset of hearing through electrical stimulation during childhood. We hypothesize that spatial hearing abilities in these children emerge with ongoing experience after CI activation. We tested this hypothesis by measuring changes in spatially relevant perceptual representations of auditory space at repeated intervals one year apart, in children who use BiCIs. In addition, a novel statistical analysis method was developed in this study, aimed at quantifying behavioral measures such that changes in spatially relevant perceptual representation of space can be better understood. The analysis produced a *localization sensitivity index*, which was utilized to delineate changes in response patterns with experience. To the authors’ knowledge, this is the first attempt to represent perceptual mapping of acoustic space and compare between the mapping patterns seen in children with BiCIs and children with NH.

## Materials and Methods

### Ethics Statement

The Health Sciences Human Subjects Institutional Review Board of the University of Wisconsin-Madison approved the work conducted here. We obtained informed consent from the parent or legal guardians on behalf of the minors/children enrolled in this study. Consent was written. In addition, children ages 7 and older signed a written assent form. Consent was recorded and documented through written forms. The ethics committees/IRB approved this consent procedure.

### Subjects

#### Children with BiCIs

Nineteen children with bilateral profound hearing loss participated. At the time of initial testing their ages ranged from 4–9 years (average age 6.5 years), and the amount of experience with BiCIs ranged from 13–51 months (average age 30 months). The aim of this study was to test the development of localization with BiCIs experience, though these children had different amounts of monaural hearing experience. Children with BiCIs were tested at repeated intervals, at least twice, with intervals separated by approximately12 months. The majority of children in this study (N = 14) were tested twice. In addition, data are shown for 4 children who participated in testing three times and 1 child who participated four times. Experimental conditions were matched between tests so that changes in performance would reflect changes in each child’s ability to perform the task. Subject demographics, as well as details regarding the age and amount of bilateral experience at time of each testing interval are listed in Table 1.

**Table 1:** Subject demographics.

Participant	Sex	Etiology	Age at Visit (yrs; mos)				Age at first CI (yrs;mos)	Unilateral experience (yrs; mos)	Age at second CI (yrs; mos)	BICI experience (mos)				First CI (Device, processor, ear)	Second CI (Device, processor, ear)
			V1	V2	V3	V4				V1	V2	V3	V4		
CIAW	M	CMV	7;8	8;7	11;10		1;2	4;3	5;5	26	37	78		N24, Freedom, R	Freedom, Freedom, L
CIAY	M	Unknown, progressive^a^	9;2	11;8			5;2	0;10	6;0	38	68			N24, Freedom, R	Freedom, Freedom, L
CIBU	M	Connexin 26	6;3	7;3	8;3		1;2	3;11	5;1	13	26	37		V1: Combi40+, Tempo+, L; V2: Combi40+, Opus 2, L; V3: Combi40+, Opus2, L	V1: Pulsarci100, Tempo+, R; V2: Pulsarci100, Opus2, R; V3: SONATAti100, Opus2, R
CIBW	F	Connexin 26	5;10	7;0			1;1	2;8	3;9	25	39			N24, Freedom, R	Freedom, Freedom, L
CICA	M	Unknown	5;6	6;6			2;5	0;0	2;5	38	49			Pulsarci100, Opus 2, simultaneous	Pulsarci100, Opus 2, simultaneous
CICF	F	Bacterial Meningitis^a^	5;6	6;7			1;6	0;10	2;4	38	50			Freedom, Freedom, R	Freedom, Freedom, L
CICL	M	Connexin 26	4;10	6;0			1;5	1;4	2;9	24	38			Freedom, Freedom, R	Freedom, Freedom, L
CICY	M	Unknown	6;9	8;0			1;0	3;8	4;8	24	39			HiRes90K/HiFocus, Harmony, R	HiRes90K/HiFocus, Harmony, L
CIDJ	F	Genetic, progressive	7;1	8;1	8;11	10;6	1;8	3;5	5;1	24	36	47	65	N24, Freedom, R	N24, Freedom, L
CIDP	F	Connexin 26	4;11	5;10			0;11	1;10	2;9	26	37			V1: Combi40+, Tempo+, L; V2: Combi40+, Opus 2, L	V1: Pulsarci100, Tempo+, R; V2: Pulsarci100, Opus2, R
CIDQ	F	Unknown	6;7	7;8	8;8		0;9	3;6	4;3	27	40	51		V1: N24, SPrint, R; V2: N24, Sprint, R; V3: N24, Freedom, R	V1: Freedom, Freedom, L; V2: Freedom, Freedom, L; V3: Freedom, Freedom, L
CIDW	M	Unknown	7;0	8;0			2;3	2;9	5;0	24	36			HiRes90K/HiFocus, Harmony, R	HiRes90K/HiFocus, Harmony, L
CIEB	F	Pendred’s, progressive	8;3	11;3			3;7	0;5	4;0	51	86			N24, Freedom, R	N24, Freedom, L
CIEC	M	Pendred’s, progressive	6;11	10;1			2;5	0;5	2;10	50	85			N24, Freedom, R	N24, Freedom, L
CIEE	M	EVA progressive	6;2	7;2			2;10	1;1	3;11	27	38			V1: Freedom, Freedom, R; V2: Freedom, N5, R	V1: Freedom, Freedom, L; V2: Freedom, N5, L
CIEF	F	Unknown	6;10	7;11			1;3	3;7	4;10	24	37			N24, Freedom, R	Freedom, Freedom, L
CIEH	M	Genetic	4;1	5;0	6;2		1;1	0;0	1;1	36	47	61		Freedom, Freedom, simultaneous	Freedom, Freedom, simultaneous
CIEK	F	EVAS^a^	7;4	8;4			4;11	0;4	5;3	26	37			HiRes90K/HiFocus, Harmony, L	HiRes90K/HiFocus, Harmony, R
CIET	M	EVAS^a^	6;11	7;11			4;10	0;0	4;10	25	37			SONATAti100, Opus 2, simultaneous	SONATAti100, Opus 2, simultaneous

^a^Children who have a history of acoustic experience

#### Children with NH

For comparison, 6 typically developing, 5-year-old NH children (5.01±0.23 years) participated in this study. Their performance was treated as being representative of that seen in the general population of NH children with ages equivalent to those of the youngest participants in the BiCIs group [11]. The NH children had no history of hearing loss, middle ear problems, or other developmental delays.

### Experimental set-up

Testing was conducted in a double-walled sound-attenuating booth (IAC) containing a horizontal semicircular array of 15 loudspeakers. The loudspeakers were positioned at ear level, approximately 1.2 meters from the listener, spanning from -70° to 70° in 10° intervals. Stimuli consisted of bi-syllabic Children’s Spondees that have been previously used for studies on spatial unmasking of speech in children with NH [14], and with BiCIs [15], recorded with a male voice at a sampling rate of 44 kHz. The speech corpus had 25 words. On each trial one of the words was randomly chosen from the list, with replacement. Thus, on any given trial the stimulus was unpredictable, thereby reducing the availability of reliable spectral cues that might be used in the computation of sound source location. This novel stimulus design was different from a previous study in this lab [11], which used a single spondaic word (“baseball”) on all trials. Stimuli were balanced for loudness using equal root-mean-square levels for all words. Stimuli were amplified and sent to the loudspeakers via Tucker Davis Technologies System III hardware. Stimulus levels averaged 60 dB SPL, with a random variation between 56 and 64 dB SPL (±4 dB) from trial to trial to minimize the availability of overall level cues.

### Experimental procedure

Testing was conducted using a 15-alternative forced choice (AFC) procedure. Stimuli were presented in random order from the loudspeakers, for a total of 10 presentations from each location, so that testing consisted of 150 total trials. Typically, children took breaks every 20 minutes, and testing was interspersed with testing on other tasks (e.g., speech perception or language measures) conducted as a part of other experiments. A computer monitor positioned at 0° had a display with 15 icons in a semi-circular array, arranged in a similar fashion to the actual loudspeakers in the room. Icons positioned underneath each loudspeaker matched icons arranged on the computer screen. On each trial the child was instructed to face forward during stimulus presentation, and to then use the mouse/monitor interface to select the icon that matched the perceived source position. After each response, children received visual feedback in the form of blinking of the correct location icon on the computer screen.

### Data Analysis

#### RMS error

Calculating the RMS error between the azimuth of the stimulus location and the listener’s responses over all 150 trials assessed localization performance in the 15-AFC sound source identification task. This was done using the following formula:

$$RMS=\sqrt{\frac{1}{K}\sum_{k=1}^{k}\frac{1}{N_{k}}\sum_{i=1}^{N_{k}}{\left(r_{k,i}-s_{k}\right)}^{2}}$$ (1)

Where *K* is the total number of sound sources, *N*_*k*_ is the number of trials at *k*th source, *r*_*k*,*i*_ is the listener’s response to the *i*th trial on which source is presented. For each trial, the difference between response and stimulus reflects the localization accuracy. However, for repeated trials at a given stimulus location, the RMS error includes both constant and variability error[13], which are determined by localization accuracy and reliability.

#### Localization sensitivity

For comparing two response groups to each pair of locations in the loudspeaker array, the p-values were calculated from the non-parametric Kruskal-Wallis test between the two response groups via the MATLAB *Kruskal wallis* function. T*he Kruskal-Wal*lis test was selected because it is analogue to a one-way ANOVA test, and can be extended to comparisons amongst more than two groups, which is a future research direction on categorical localization. Hence results of analyses from the current study can be directly compared to those from future work. Each p-value was transformed to a z-score, which is defined as the localization sensitivity index (LSI). This analysis produces a matrix of LSIs in coordinates of response locations. For N = 15 target locations, the total number of LSI values was 105 [15*(15–1)/2 = 105]. For example, the results shown in Fig 1 resulted from calculation of the LSI matrix.

Note that the scatter plots of the response distributions (from which RMS is computed) and the LSI matrix reflect different aspects of the data. While the response distribution scatter reflects the absolute dependence of the responses upon the stimulus locations, the LSI matrix reflects discrimination ability, which compares responses between any pair of stimulus locations. The latter has the benefit of quantifying how differently subjects respond to any given pair of stimuli. This analysis also has the advantage of producing a response matrix that describes the types of localization errors that subjects make. The matrix is symmetrical about the diagonal. The matrix was interpolated via the MATLAB *contour* function to find a contour of LSI with a z score of 1.65 (the 95^th^ confidence level as the statistical threshold indicative of two locations being perceptually differentiated). We defined the contour of LSI = 1.65 as the sensitivity threshold. The overall localization sensitivity (LS) was characterized for individuals by averaging LSIs in the LSI matrix, which reflects the overall spatial mapping ability. Fig 1C and 1D show the LSI matrix for the two examples. While the two examples yielded similar RMS error values, they displayed different patterns of LSI. Example II (Fig 1D) resulted in higher overall LS.

There are several advantages to using this non-parametric approach to examine the sound localization ability of CI users. First, a non-parametric test compares the median values of the data sets, without requiring calculation of the means. This is important because there are an insufficient number of trials per location to accurately estimate the mean and standard deviation of the responses. Thus a non-parametric test is appropriate. Note that the comparison of LSI across studies is only suitable for data with same number of repetition trials. Second, while a one-way ANOVA test may yield an inaccurate estimate for data with non-normal distribution, the Kruskal-Wallis non-parametric approach does not require such a-priori assumptions. This is of particular importance in the present study. For adult listeners with NH, it is reasonable to assume a normally distributed representation of sound sources [13] because the responses at each location are likely to be stable and consistent. However, the response distribution for a source location collected from children who experienced auditory deprivation prior to being bilaterally implanted is less likely to follow a normal distribution.

## Results

### Localization Root Mean Square (RMS) Error Calculations

**Fig 2:**
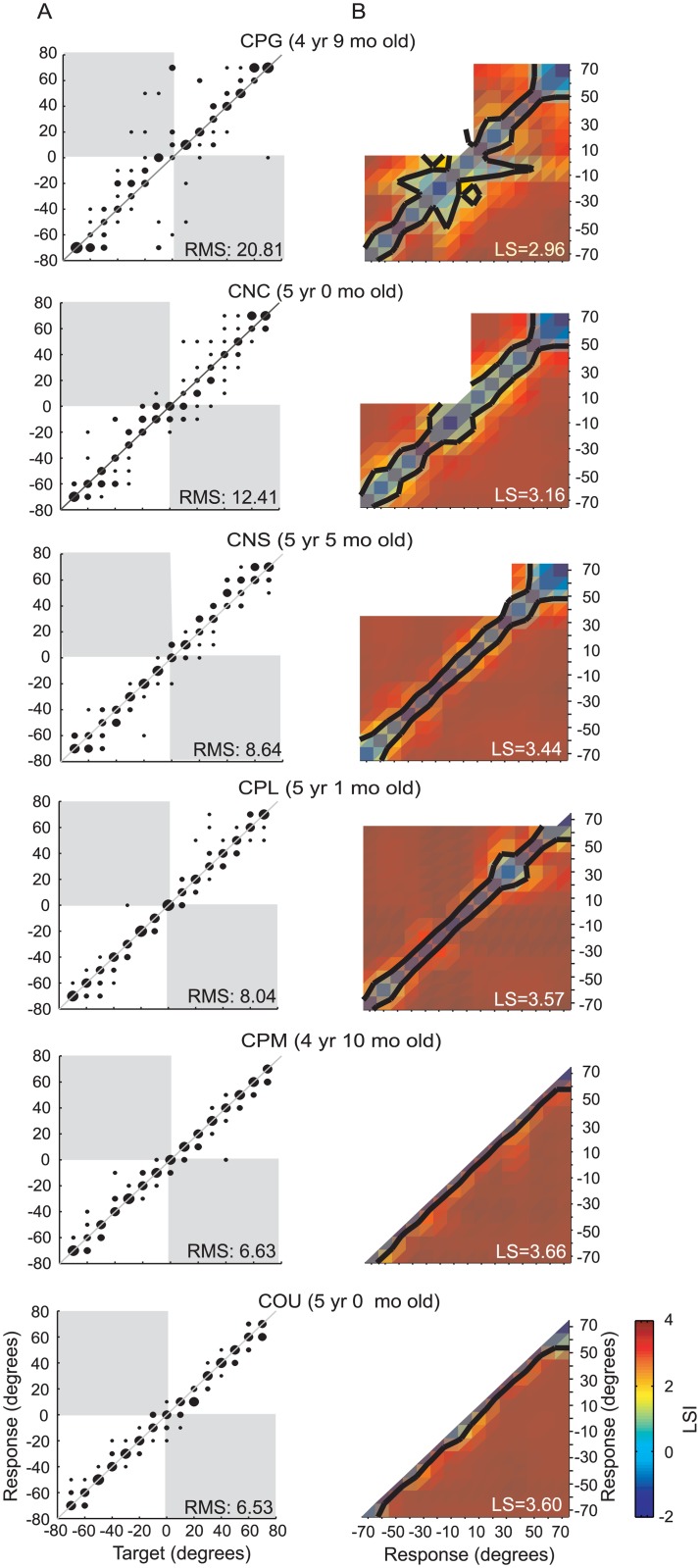
Results from the NH subject group. (A) Scatter plots for sound source identification with the 15-loudspeaker array in 6 individual NH subjects. From top to bottom, subjects are ordered by the RMS errors from largest to smallest values. (B) The LSI matrix displays spatial mapping representations from individual subjects in (A).

Fig 2 illustrates localization performance in the 15-AFC identification task for the six NH children. In Fig 2A, stimulus-response scatter plots show the proportion of each response (in degrees azimuth) as a function of the 15 target locations. Within each panel, the inset shows the RMS error values. Children in the NH group predictably displayed stimulus-driven responses along the diagonal, and distributed their response in a continuous manner along the location dimension, spanning -70° to 70°.

**Fig 3:**
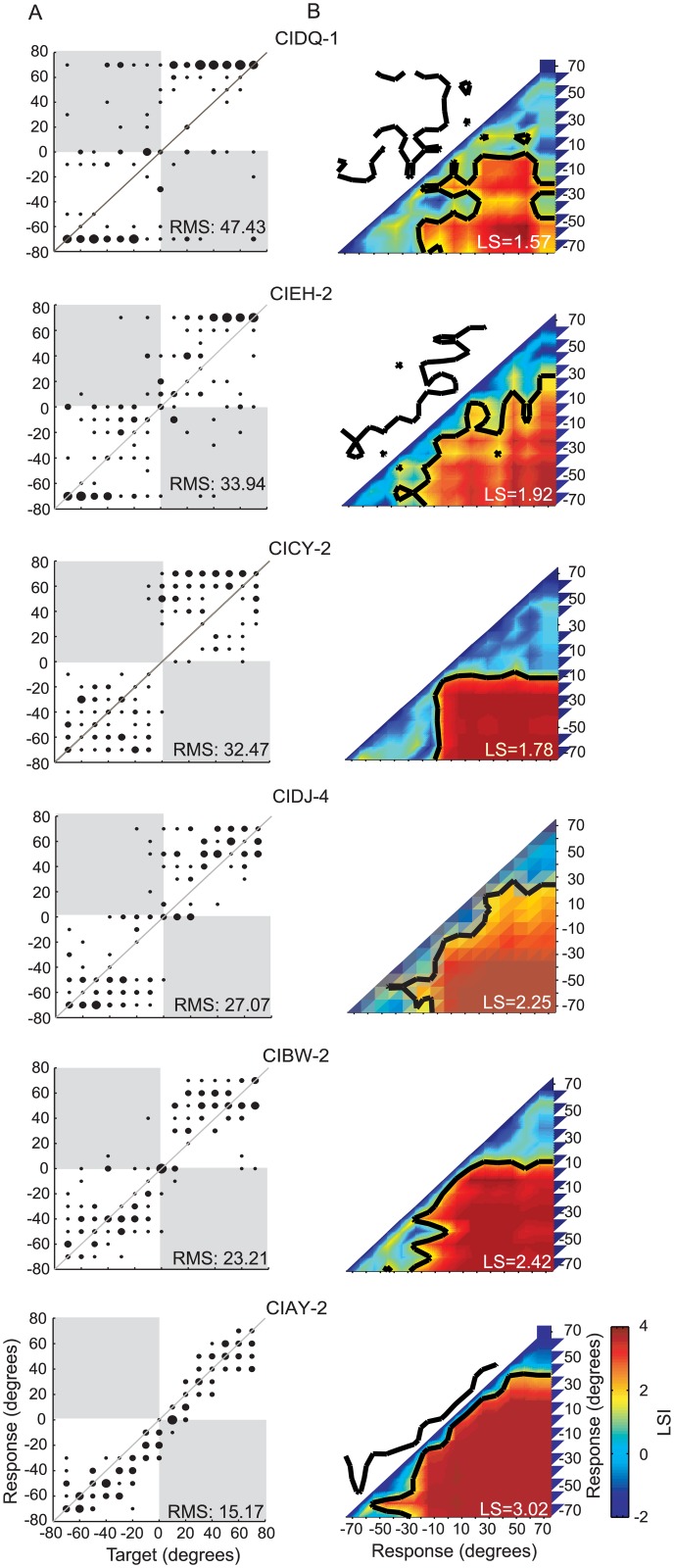
Typical examples in the BiCI group. (A, B) Same as Fig 1.Participant codes are in the format CI*XX-x*, *with—number* as the order of testing interval. From top to bottom, the subjects are ordered by RMS errors from largest to smallest values.

Results and RMS values are shown in Fig 3A for six children with BiCIs. Data from these children are arranged according to RMS error (worse near the top and best near the bottom). The results are clearly different in this group than in the NH group. A notable feature of the data in Fig 3A is the response patterns that can be characterized as “clustered” or “scattered” along the source location dimension. These data were selected to illustrate typical response types observed in this participant group, and to point out the fact that different patterns of errors may not necessarily be distinguished with the RMS calculation. For example, data for subjects CIEH-2 and CICY-2 had nearly equivalent estimates of localization accuracy as measured by RMS error calculations (CICY-2: RMS = 32.45°; CIEH-2: RMS = 33.94°). However, upon visual examination of the response patterns, CIEH-2 showed variable, “scattered” responses with large deviation in the region between -40° to 40°. In contrast, subject CICY-2 demonstrated a different type of pattern; there were “clustered” responses in the left and right regions with very few responses at 0°. Such discontinuous “clustered” responses appeared in different formations in the BiCIs group. For example, subject CIDQ-1 chose between two extreme locations of -70° and 70° (see Fig 3 top panel). In addition, this subject had numerous left/right errors and a small number of responses that fell along the diagonal line (line of unity representing perfect correspondence between target locations and response options). This type of response distribution resulted in a large RMS error of 47.4°. Subjects CIDJ-4 and CIBW-2 showed “clustered” responses to the left and right but had a response preference to 0° when the target location was in the region of -20°~20°. Finally, CIAY-2 provides an example of responses similar to those obtained from the children in the NH group, especially for locations between -40° and 50°, with evenly distributed responses along the diagonal. The ranges of RMS errors for all individual data, from all children tested overlapped for the two groups (NH: 6.5°-20.8°; BiCIs: 13.8°-47.6°). The means ± SD were, on average, smaller in the NH group (NH: 10.5°±8.5°, BiCIs: 28.5° ± 7.9°).An aspect of the data that is not captured by the RMS values, however, is the notable manner by which children with BiCIs tend to cluster or categorize their judgments of sound source locations. This will be discussed further as we present the localization sensitivity measure.

### Localization Sensitivity (LS) Calculations

In Figs 2B and 3B, results from the LS calculations are shown as a matrix, thus illustrating for each subject the perceptually mapped representation of acoustic space. The LSI is color-coded, such that “cold colors” (blue) represent areas of indiscriminable response groups, and “hot colors” (red) represent areas with highly discriminable response groups. The LSI colormap was produced with a linear interpolation using a MATLAB *interp* function. The black contour represents location pairs that met the 95th confidence (corresponding to an LSI value, or z-score, of 1.65) for likelihood of belonging to different response distributions. This confidence level was implemented as the statistically significant threshold for indicating that two locations were perceptually differentiated. LS values for each subject are shown in the inset of each panel in Figs 2B and 3B.

The spatial mapping representations were distinctly different when comparing results from children in the NH and BiCIs groups. Children in the NH group (Fig 2B) displayed a narrow but continuous “belt-shaped” region of indiscriminable response groups along the diagonal; however, all other response groups were highly discriminable. In contrast, children in the BiCIs group (Fig 3B) demonstrated responses with notably larger indiscriminable clusters, reflecting many locations in space that are coarsely mapped. Clusters at the top/right and the bottom/left quadrants of the LSI matrix reflect localization confusion among locations on the same side.

Compared with the NH children whose overall performance generally showed high LS (2.95–3.67, mean ± SD: 3.40±0.28), children in the BiCIs group had LS that were much lower (0.89–3.10, mean ± SD: 2.23±0.46). The response types were classified into 5 patterns that are described in the next section. Results suggest that NH 5-year-old children are able to map the acoustic space in the frontal horizontal plane on a continuum with fine resolution. In contrast children with BiCIs are more likely to divide the acoustic space into left and right, and where finer resolution is observed it emerges from the fact that they tend to make categorical judgments regarding auditory space, rather than identifying target locations with fine resolution. These results led us to investigate whether, with additional experience, children with BiCIs develop improved localization strategies and are able to transition from more coarsely based response types to more fine-grained localization strategies over time.

### Longitudinal results of localization sensitivity

**Fig 4:**
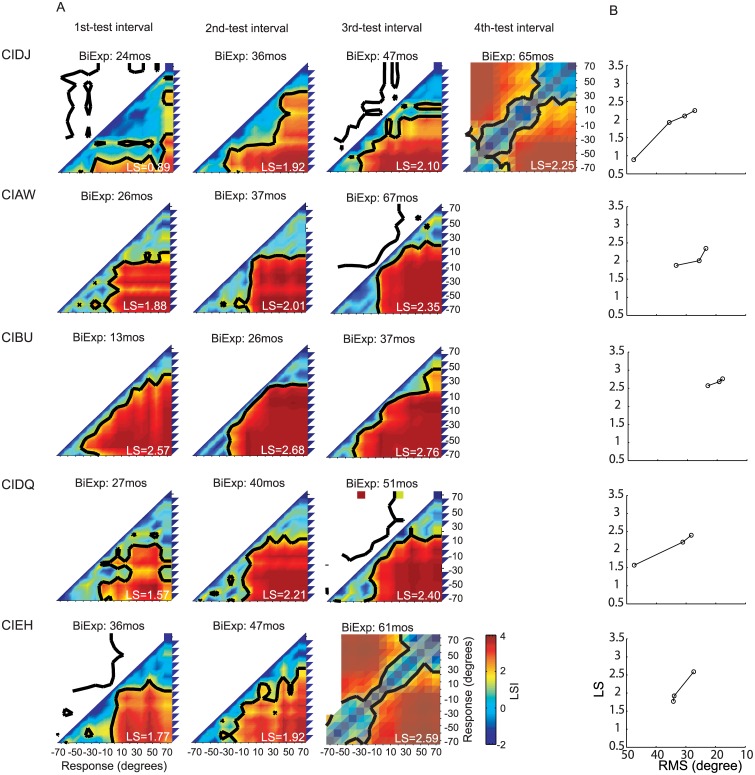
Individual performance in the sound source identification task for 5 children in the BiCI group with more than 2 test intervals. (A) Each row shows the LSI matrix for spatial mapping representation for each child at each testing interval. (B) Changes in localization abilities at each testing interval for each child. Values are shown on the Y-axis for localization sensitivity and on the X-axis for RMS errors.

**Fig 5:**
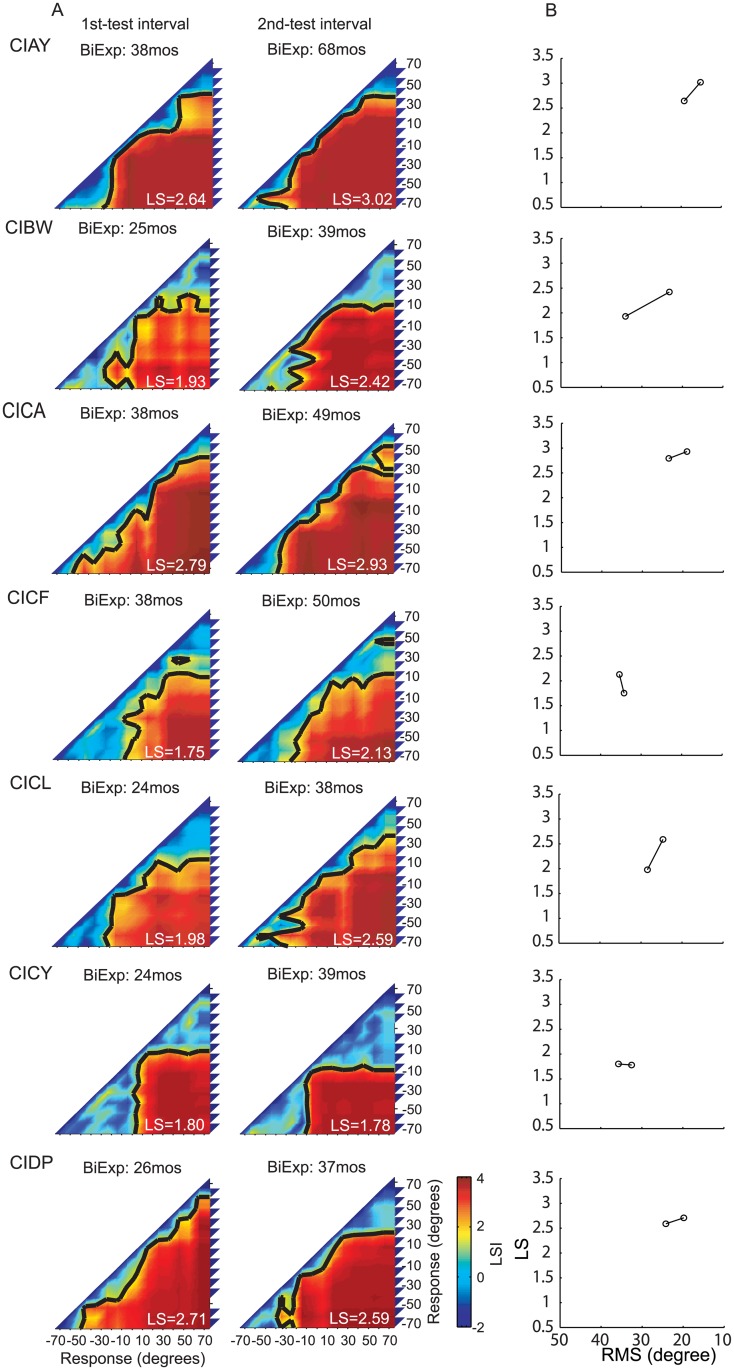
Individual performance in the sound source identification task for 7 children in the BiCI group with 2 test intervals. Same as Fig 4.

**Fig 6:**
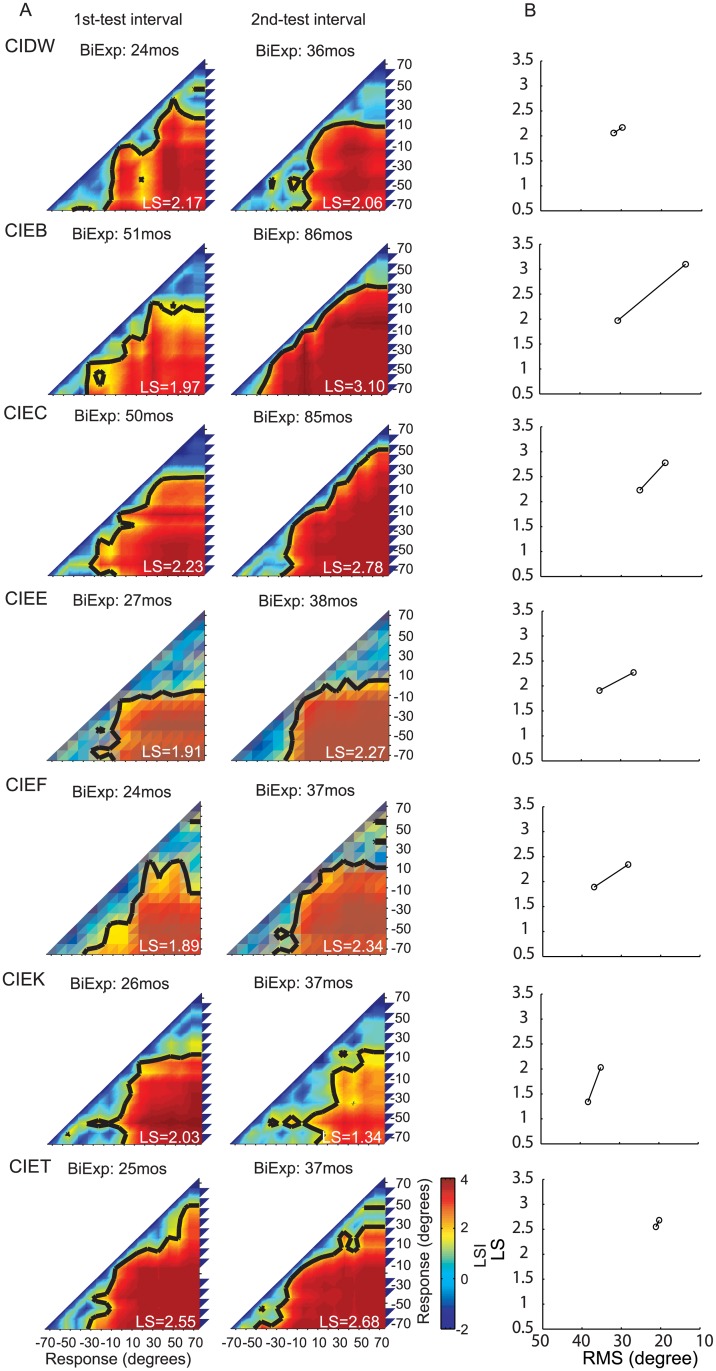
Individual performance in the sound source identification task for 7 children in the BiCI group with 2 test intervals. Same as Fig 4.

Longitudinal results are shown in Figs 4–6. LSI matrices reveal the localization spatial mapping representations and can be used to model responses and to classify the response patterns. To examine the changes of localization strategies with bilateral experience, five different LSI-based response models were constructed according to well-defined criterion below (categories Type I-V). Each response model was constructed with the simulated responses to 15 target locations for 15 possible response locations from -70° to 70° in 10° intervals. At each target location there were 10individual simulated responses, and therefore for each model an amount of 150simulated responses was used to generate a response matrix by keeping the same numbers and range of responses as that of the actual response. The LSI matrix and sensitivity thresholds of the model responses were calculated, using the same procedure as that employed for obtaining sensitivity thresholdsof the real responses (see Method).

Type I: NH-like narrow belt shape, with an ability to discriminate locations separated <20°. In this model, simulated responses were produced through uniform distribution within ±10° of each target location.
Type II: Center belt shape, with an ability to discriminate locations in the center region (-20°to 20°) but with lack of ability to discriminate between locations in the lateral regions. In this model, simulated responses were generated with a uniform distribution of responses within ±10° of each target location, when the targets were between -30° and 30°. In addition, for this model, when the targets were +30° to +70°, responses were distributed uniformly within that range, and for targets at -30° to -70° responses were uniformly distributed within the range of -30° to -70°.
Type III: Broad belt shape, with coarse resolution. In this model, simulated responses were generated by applying a uniform distribution within ±30° of each target location.
Type IV: Left-right categorical shape, with an ability to categorize locations to the left and right, but inability to discriminate locations within the same hemifield. In this model, simulated responses were produced with a uniform distribution in the range of 0° to -70° when the target was on the left and 0° to +70° when the target was on the right.
Type V: Random response shape, inability to localize or categorize sound source locations. Responses were randomized completely in the range of target locations spanning from -70° to-+70°.

The purpose of generating these five categories was to enable us to determine how localization abilities of BiCIs users change with experience. Thus, results from each child, at each testing interval, were analyzed to generate response LSI matrices (Figs 4–6). The response LSI matrix was then normalized by its peak and compared to the five normalized LSI-based model matrices, and classified into one of the five categories by means of a minimum mean square error (S1 Table).

Table 2 shows the changes in matrix classifications, with increasing bilateral listening experience, for each child in the BiCIs group. Overall, 2/19 children reached the precision demonstrated by Type I (best) responses for a minimum of one of the testing intervals. An additional group of children (12/19), with at least 36 months of bilateral listening experience demonstrated Type II responses. Of these children, 10 traversed through the worse-performance types at earlier testing intervals, and 2of those 10transitioned from Type IV/V to Type II with about 1 year of additional experience (CIDQ, CIBW). Four of the children who participated twice showed no improvement at the second testing interval: One showed Type II responses at both testing intervals (CIEC) and one showed Type III responses at both testing intervals (CICF); Finally, two children who were tested twice remained in the Type IV category (CICY, CIEE). These data suggest that, in general, 24 months BiCIs experience is associated with the ability to categorize locations to the left and right, however, >24 months of BiCIs experience seems necessary to generate more fine-grained location identification strategies (Types III, II and I).

**Table 2:** Changes of localization patterns with increasing BiCIs experience.

	Type V	Type IV	Type III	Type II	Type I
CIDJ	1(24mos)		2(36mos); 3(47mos); 4(65mos)		
CIAW		1(26mos); 2(37mos)		3(67mos)	
CIBU			1(13mos)	2(26mos)	3(37mos)
CIDQ		1(27mos); 2(40mos)		3(51mos)	
CIEH		1(36mos)	2(47mos); 3(61mos)		
CIAY			1(38mos)	2(68mos)	
CIBW		1(25mos)		2(39mos)	
CICA			1(38mos)	2(49mos)	
CICF			1(38mos); 2(50mos)		
CICL			1(24mos)	2(38mos)	
CICY		1(24mos); 2(39mos)			
CIDP			1(26mos)	2(37mos)	
CIDW			1(24mos)	2(36mos)	
CIEB			1(51mos)		2(86mos)
CIEC				1(50mos); 2(85mos)	
CIEE		1(27mos); 2(38mos)			
CIEF			1(24mos)	2(37mos)	
CIEK		1(26mos)	2(37mos)		
CIET			1(25mos)	2(37mos)	

The numbers in the table indicate the testing interval, and the numbers in parentheses show the months of bilateral hearing experience.

**Fig 7:**
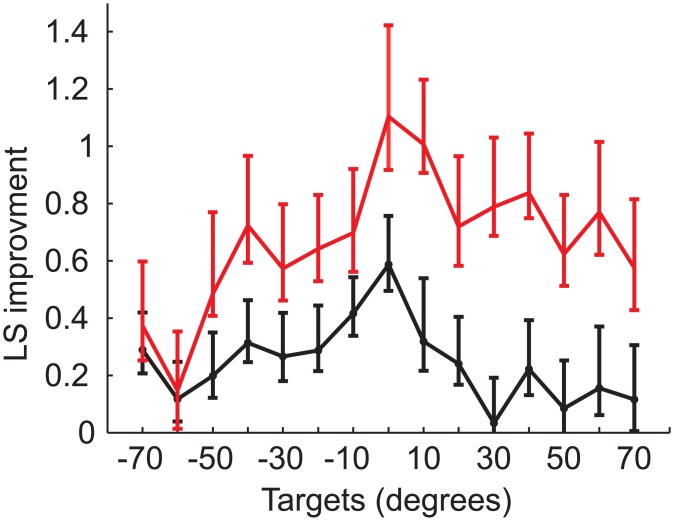
Improvement in localization sensitivity per target location. Population improvement is presented as the difference in localization sensitivity between two tests. Error bars indicate bootstrap bias-corrected and accelerated confidence intervals (p<0.05). Black curve indicates the improvement over one year, and red curve includes the improvement with more than one year.

Each response matrix was also analyzed to produce LSI values (as previously described). Overall increase (improvement) in average LSI values per target location for the population of children tested is shown in Fig 7. The greatest improvement was evident in the central region of the loudspeaker array (-30° to 20°), with a mean increase of 0.2 in LS per target for test intervals 1 year apart (Fig 7, *black* curve). When comparing testing intervals >1.5 years apart (Fig 7, *red* curve), changes in overall LS were significantly greater than the one-year interval changes (t-test, p<0.01). The greater change observed for larger testing intervals implies that, with an increasing amount of bilateral hearing experience, sound localization continues to improve beyond the improvement seen in the 1-year interval.

**Fig 8:**
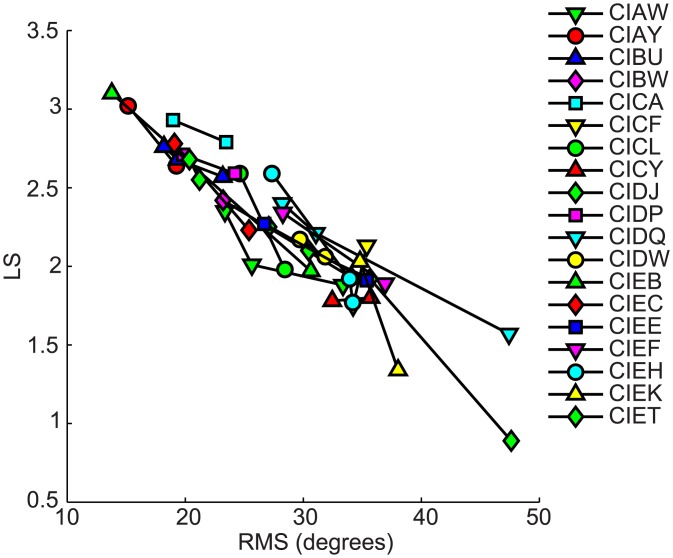
Changes in localization abilities at each testing interval for each child in the BiCI group. Each line represents data from a single child at various testing intervals. Values are shown on the Y-axis for localization sensitivity and on the X-axis for RMS errors.

Whereas the RMS is determined by both localization accuracy and response precision, LS is only dependent on differences of response distributions to different stimulus locations. In other words, LS does not reflect stimulus-response correlations, but rather it reflects a spatial mapping ability for different locations in the continuous acoustic space. In contrast, RMS does reflect the dependence of responses on stimulus locations, but it provides no information about how subjects map acoustic space to spatially-relevant perceptual representations. The merits and limitations of the LS and RMS approaches as described henceforth, suggest the utility of an integrated approach that presents LS coupled with RMS data for a more complete picture of the perceptual spatial representations. Table 3 displays all RMS and LS values and Figs 4B–6B show the dependence of LS upon RMS for each testing interval for each subject. Though for most subjects both RMS and LS improved between testing intervals, improvement in RMS was not directly predicted from LS. In other words, RMS did not change in a fixed proportion relative to improvement detected with the LS analysis, which can be observed in the different slopes of the lines connecting the intervals for each subject. Fig 8 shows the overall correlation between RMS and LS (spearman correlation r = -0.93, p<0.001). The changes across testing intervals in RMS and LS were correlated but this correlation was not perfect (spearman correlation, r = -0.77, p<0.001).

**Table 3:** RMS and LS values for each subject at each testing interval.

	RMS	LS
**NH group**		
COU	6.53	3.60
CPM	6.63	3.67
CPL	8.04	3.57
CNS	8.64	3.44
CNC	12.41	3.16
CPG	8.76	2.95
**BiCIs group**		
CIAW—1	33.36	1.88
CIAW—2	25.65	2.01
CIAW—3	23.37	2.35
CIAY—1	19.25	2.64
CIAY—2	15.17	3.02
CIBU—1	23.18	2.57
CIBU—2	19.36	2.68
CIBU—3	18.20	2.76
CIBW—1	34.07	1.93
CIBW—2	23.21	2.42
CICA—1	23.47	2.79
CICA—2	18.97	2.93
CICF—1	34.22	1.75
CICF—2	35.37	2.13
CICL—1	28.44	1.98
CICL—2	24.63	2.59
CICY—1	35.70	1.80
CICY—2	32.46	1.78
CIDJ—1	47.62	0.89
CIDJ—2	35.66	1.92
CIDJ—3	30.46	2.10
CIDJ—4	27.07	2.25
CIDP—1	19.83	2.71
CIDP—2	24.21	2.59
CIDQ—1	47.43	1.57
CIDQ—2	31.07	2.21
CIDQ—3	28.23	2.40
CIDW-1	29.72	2.17
CIDW-2	31.86	2.06
CIEB-1	30.64	1.97
CIEB-2	13.78	3.10
CIEC-1	25.40	2.23
CIEC-2	19.08	2.78
CIEE-1	35.38	1.91
CIEE-2	26.70	2.27
CIEF-1	36.93	1.89
CIEF-2	28.27	2.34
CIEH—1	34.20	1.77
CIEH—2	33.94	1.92
CIEH—3	27.35	2.59
CIEK-1	34.80	2.03
CIEK-2	38.04	1.34
CIET-1	21.21	2.55
CIET-2	20.36	2.68

In summary, children who experience early auditory deprivation and are fitted with BiCIs demonstrated significant improvement on sound-source localization sensitivity with experience. Many of the children used a categorical strategy to judge the locations of sound sources, and typically perceived sounds as emanating from the extreme locations to the left and right. With experience, greater localization sensitivity emerged first in the central region of the spatially distributed target sources, and subsequently extended to lateral positions. Auditory experience with BiCIs could possibly lead to the refinement of localization strategies. In addition, the analyses developed in this study to demarcate amongst 5 different response pattern types, together with the LS approach, provide a potentially more useful means of describing spatial mapping of sound compared with more traditional methods that focus on RMS errors. This analysis approach can facilitate the tracking of localization changes over time in individual subjects in a way that provides information about how they are mapping sound to space, rather than only focusing on their overall error rates.

## Discussion

### Experience-dependent emergence of spatial hearing

The present study focused on emergence of sound localization skills in children with BiCIs. Results showed that changes in spatial hearing show refinement, from categorical perception to more fine-grained perceptual mapping of auditory space. In addition, for most children, improved localization occurred with an increase in bilateral hearing experience. Our major finding, that localization sensitivity improvement is seen through the emergence of perceptual mapping, shows that changes are not uniform across listeners. In addition, changes within listeners are not uniform across all locations. Rather, improvement occurs first in the central region of acoustic space, and subsequently for lateral positions. This may be accounted for by the fact that the most frequently encountered stimuli and experiences with auditory targets are in the frontal (central) region of space, where people generally tend to bring objects of interest into focus. In general, neural encoding is most precise for frontal locations [16]. Thus, in relation to our data, children with less bilateral hearing experience were able to categorize locations to the left and right, an ability that likely requires rudimentary processing of auditory inputs. An additional factor is that spatial cues such as interaural level differences are poorly represented by CI clinical processors at lateral positions [17] thus BiCIs users are more likely to be able to learn how to utilize cues related to central regions than lateral regions.

Experience plays an important role in sound localization improvement in children with CIs. The auditory structure involved in higher-order processing of spatial cues, the auditory cortex, has a remarkable capacity for plasticity and plays an essential role in experience-induced plasticity [18]. Research with non-human animal species provides evidence that deafness during the neonatal and extended periods beyond, results in loss of normal cochleotopic organization. In addition, prolonged auditory deprivation leads to a dysfunctional intrinsic cortical circuitry and multimodal reorganization of primary cortex [18–20]. This reorganization may reduce intrinsic responses of neurons in auditory cortex, and eliminate descending projections to the midbrain, with consequences on spatial sensitivity of neurons in the inferior colliculus [21]. The issue to consider regarding BiCIs users is that binaural cues are not received by the auditory system of these individuals, at least not with fidelity. One of the main factors is the fact that the CI processors in the two ears act independently, thus producing interaural time difference (ITD) cues that are absent or spurious. In the event that engineering efforts yield successful restoration of ITDs to BiCIs cues (for review, see [22]), children who are currently using BiCIs will not be hearing stimuli with ITDs until a later stage in development. We consider the possibility that plasticity issues may place constraints on whether spatial remapping that will improve localization can take place following deprivation-induced reorganization.

Another important issue to consider is that the children with sequentially implanted BiCIs are likely to have asymmetrical or spatially unbalanced responses between the two populations of ipsilateral and contralateral neurons feeding into the auditory pathway. Sequential stimulation of the auditory pathway has been shown to promote a functionally asymmetric central auditory pathway with reduced spatial selectivity for sound sources [23, 24]. Unilateral dominance in sequential implantation has also been reported in the cat [25]. Children with a prolonged unilateral CI experience may acquire spatial hearing abilities that are not congruent across different levels in the auditory pathway. While physiological measurements in humans have suggested that bilateral auditory inputs can protect the brain from unilaterally-driven reorganization [26], behavioral evidence to support that notion is currently not available. Behavioral data in this study showed that some children have limited localization ability, that is, they seem to be restricting their responses to categorical judgments about sound source locations. This limitation might be the result of the unilaterally-driven activation described in physiological studies, and might have resulted from exposure to a unilateral CI before activation of CIs in both ears [23–26]. Most children with BiCIs sin this study underwent a period of unilateral activation (mean = 22 months; range 0–51 months) prior to receiving the second CI. However, there did not appear to be a dependence of localization on unilateral experience. It may be the case that by the time children were tested at the first interval they already had significant amounts of bilateral experience. Nonetheless, the fact that they continued to improve with additional experience, which is the focus of this study, suggests that localization in children with BiCIs involves an emerging and dynamic process.

In several of the children in the BiCIs group, an interesting observation was made regarding their behavioral responses to the auditory stimulus. Following stimulus presentation, some subjects initially reflexively turned their head to the correct side of a sound source, but then selected a sound in the opposite hemifield (verbally or via pointing), essentially resulting in errors. The nature of the task was such that sound localization was assessed on the basis of decision-making about source locations via approach-to-target behavior, rather than the head orienting behavior. The conflict between reflexive head orientation and the verbal/pointing decision, as per instructions, may be rooted in the different roles that are played by the lower circuits (brainstem and midbrain) and by more central mechanisms (auditory cortex and other cortical areas) involved in the sound localization process. Put together, this longitudinal behavior study on children with BiCIs and the previous physiological and behavioral animal studies imply that the experience-dependent factor is likely to be involved in the emergence, preservation and development of spatial hearing.

### Neuronal mechanisms of localization

The finding that sound localization varies across children, and also that within children longitudinal changes are observed, leads us to consider what is known about the neural mechanisms involved in sound localization in mammals. Two main hypotheses have been proposed to explain how the auditory system of mammals encodes auditory space. The topographical place code idea assumes that particular locations in auditory space are represented by neural activity for specific neurons, arising through neuronal computations of interaural time difference (ITD) and interaural level difference (ILD) [27, 28]. Alternatively, the population rate code idea, in which sound-source locations are represented by differences between two broadly tuned, contralaterally- and ipsilaterally- preferring neurons, has been suggested for the auditory midbrain [29] and also for auditory cortex [30, 31]. The population of children studied here provides data that can potentially be accounted for by a hybrid of these mechanisms. The children in this study were unlikely to receive ITD cues that were reliable and that represented physiologically relevant acoustic space through their clinical processors. In addition, the implanted electrode arrays were generally inserted into the basal and middle regions of the cochlea; little if any direct stimulation occurred in the apical regions of the cochlea where neurons are tuned to low frequencies. Thus, little can be said regarding the use of ITDs in the temporal fine structure of the signal. Our conclusions must be based on possibilities for encoding ITDs in the envelopes of high-rate signals and ILD processing, as these are the primary cues that could potentially be available to these participants.

Most children with BiCIs in this study not only displayed correct responses for left vs. right hemifields, but also demonstrated better discriminability for source locations near midline than for those in the more lateral locations. The children may have thus encoded information through a simple two-channel rate coding scheme, in which neurons encode information from spatial locations to the far left and far right, and neuronal activity with the largest rate changes near the center of the spatial tuning functions determines the side of the head to which the source is perceived [31].

Another feature observed in the localization abilities in many of the children was difficulty discriminating locations within a given hemifield, which is likely due to absence of refined coding of spatial cues. The most robust auditory cues that are known to represent the spatial dimension in the horizontal plane are the interaural time differences (ITDs) for low-frequency stimuli, and interaural level differences (ILDs) for high-frequency stimuli. Additional localization cues are provided by ITDs in the slower-modulated envelopes of high-frequency stimuli, and these are of particular relevance to CI users, whose processors preserve envelope cues but discard low-frequency fine-structure cues [32, 33]. Though coding of ITDs/ILDs occurs at the level of the brainstem, and brainstem activation is sufficient for producing voluntary orienting behavior [34, 35], the auditory cortex plays the role of mediating the orienting behavior [36] and enabling the animal to remember the sound source [37]. Ablation of the auditory cortex has resulted in an inability to initiate a localization behavior, rather than impaired localization perception [38], and more fine-grained approaches to selectively deactivating cortical responses have similarly highlighted the role of the auditory cortex in the ability of an animal to re-learn new spatial mapping cues after altered experiences [39].

### Implication of rehabilitation and training of children with cochlear implants

In this study, children with BiCIs varied in their abilities to perform tasks of absolute localization. Some children's performance was similar to that of NH children with equivalent hearing ages, after several years of bilateral hearing experience, whereas other children found the task to be difficult and displayed large errors in localization even with extended experience. The role of plasticity becomes relevant when the ability of deaf children to function in realistic acoustic environments is considered, and when bilateral implantation may play a big role in the outcomes. Previous studies in congenitally deaf children suggest that the central auditory pathways are maximally amenable to change in the first 3.5 years of life, with reduced plasticity beyond 7 years of age [40]. In the present study, six children were bilaterally activated prior to age 3.5 years, but the remaining 13 children were activated with the second CI between 3.5 to 7 years of age (see Table 1). The improvements in sound localization seen in this study were not limited to the early bilateral group. In fact, the 8–12 year old children who did not show spatial hearing acuity after 3-months of activation [2], did show improvements after 2 years of bilateral experience. These findings suggest that, while early activation may result in more rapid acquisition of spatial hearing abilities in some children, acquisition of spatial hearing skills can occur into late childhood and the teenage years [9]. The individuals in the current study displayed different improvements with age and bilateral experience. The extent to which this finding reflects individual differences in how children approach the localization problem and strategize when making their decisions needs to be explored further.

Another important factor is the possibility that training-induced improvements, rather than simply added experience, could enhance sound localization performance. In NH listeners there is clear indication that training can result in improved sensitivity to ITDs and also to ILDs [41]. Also, it has been shown that humans can adaptively recalibrate and refine the use of sound localization cues [5]. Behavior studies in animals have shown remarkable localization improvement through training with feedback [42], and significant enhancement in ILD lateralization, specific to the trained cue and frequency [43]. It is possible that CI users will gain little benefits from training and recalibration found in other studies, because of the limited availability of binaural cues[44], which are not likely to be presented in a reliable manner. However, if reliable binaural cues could be presented, then, training with binaural cues could result in a strengthened association between auditory percepts and spatial locations [45].Therefore, future work may benefit from the development of binaural sound coding strategies that capture and present binaural cues with fidelity, so that auditory localization abilities of children who use BiCIs can be more similar to those of children with normal hearing.

## Conclusions

In the present study, we investigated the sound localization strategies of children with BiCIs and NH children using the 15-AFC sound source identification paradigm and the localization sensitivity analysis scheme that allowed us to describe the previously unknown perceptually mapping representation of acoustic space. Two primary observations are worth noting. First, we found that children with early auditory deprivation and less auditory experience make categorical judgments regarding sound source locations to left and right sides, whereas children in the 5-year-old NH group are able to map sound source locations with 10° resolution in the horizontal plane. Second, children with BiCIs show improved ability to perceptually map acoustic space with increasing bilateral hearing experience. Our observations suggest that children with BiCIs develop their localization strategy by transitioning from sound source categorization strategies to more fine-grained localization identification strategies.

The current status of treatment with children who are deaf is such that BiCIs have become ubiquitous, because evidence suggests their benefit over unilateral CIs for spatial hearing [3] and speech understanding in noise [15] (for review see [22, 46]). However, the extent to which they provide benefits that raise the level of performance to that seen in NH children is not well known, and the current study attempted to improve our understanding of the emergence of localization skills with increased experience. Sound localization deficits observed in CI users could be reduced by exposure to spatial hearing tasks that focus on training with reliable binaural cues.

## Supporting Information

S1 Table Mean square errors of each response matrix to each model. For each subject, the values at each visit are shown for models representing Type I, II, III, IV and V. The bold numbers in the table indicate the minimum mean square error.
(DOCX)
