# Supplementary material for: Development of Sound Localization Strategies in Children with Bilateral Cochlear Implants
Source: PLoS One. 2015 Aug 19;10(8):e0135790. doi: 10.1371/journal.pone.0135790 (PMC4545829; doi:10.1371/journal.pone.0135790)
Supplement: S1 Table — For each subject, the values at each visit are shown for models representing Type I, II, III, IV and V. The bold numbers in the table indicate the minimum mean square error. (DOCX) [file pone.0135790.s001.docx]

S1 Table. Mean square errors of each response matrix to each model

|  | **Type I** | **Type II** | **Type III** | **Type IV** | **Type V** |
| --- | --- | --- | --- | --- | --- |
| CIDJ-1  CIDJ-2  CIDJ-3  CIDJ-4 | 0.5300  0.2436  0.2132  0.1772 | 0.4967 0.2367 0.1523 0.1281 | 0.3565 **0.0823 0.0574** **0.0634** | 0.4457 0.1745 0.1466 0.1259 | **0.3244**  0.5779  0.5230  0.4965 |
| CIAW-1  CIAW-2  CIAW-3 | 0.2789  0.2787  0.2271 | 0.2085  0.1629 **0.0754** | 0.1344 0.1574 0.1100 | **0.1009**  **0.0820** 0.1087 | 0.4367  0.6551  1.1359 |
| CIBU-1  CIBU-2  CIBU-3 | 0.1069 0.1107  **0.0941** | 0.0807 **0.0674** 0.1250 | **0.0455** 0.0855 0.1106 | 0.1708 0.1897 0.1847 | 5.0706  0.6157  1.4688 |
| CIDQ-1  CIDQ-2  CIDQ-3 | 0.3404 0.2281  0.2070 | 0.2877 0.1444 **0.1048** | 0.1900 0.1169 0.1242 | **0.1583** **0.0746**  0.1445 | 0.3616  0.7720  0.9861 |
| CIEH-1  CIEH-2  CIEH-3 | 0.2917 0.2434 0.1354 | 0.2287 0.1942 0.1336 | 0.1456 **0.0847** **0.0515** | **0.1097** 0.1662 0.1502 | 0.6596  0.3969  0.6396 |
| CIAY-1  CIAY-2 | 0.1420  0.0702 | 0.1123 **0.0608** | **0.0969** 0.1428 | 0.1258 0.2661 | 0.8262  0.8944 |
| CIBW-1  CIBW-2 | 0.2489  0.1819 | 0.1380 **0.0855** | 0.1318 0.1416 | **0.1223** 0.1560 | 0.5450  0.6848 |
| CICA-1  CICA-2 | 0.0946  0.0864 | 0.0904 **0.0487** | **0.0584** 0.1060 | 0.2335 0.2022 | 0.9901  0.8417 |
| CICF-1  CICF-2 | 0.2901  0.2120 | 0.1922 0.0901 | **0.1037** **0.0607** | 0.1390 0.0969 | 0.6318  0.5682 |
| CICL-1  CICL-2 | 0.2193 0.1240 | 0.1118 **0.0871** | **0.0766** 0.1022 | 0.1393 0.1194 | 0.9053  0.6919 |
| CICY-1  CICY-2 | 0.3617 0.3897 | 0.2715 0.2649 | 0.2194 0.2606 | **0.1699** **0.1030** | 0.5378  0.6901 |
| CIDP-1  CIDP-2 | 0.0840  0.1570 | 0.0976 **0.0741** | **0.0513** 0.1034 | 0.1648 0.1917 | 1.2598  0.9815 |
| CIDW-1  CIDW-2 | 0.2071  0.2474 | 0.1366 **0.1136** | **0.1208** 0.1353 | 0.1637 0.1462 | 0.6605  0.4714 |
| CIEB-1  CIEB-2 | 0.2270  **0.0458** | 0.1302  0.0750 | **0.0742**  0.0948 | 0.1786  0.2188 | 0.5920  0.7401 |
| CIEC-1  CIEC-2 | 0.1997  0.0958 | **0.0845** **0.0882** | 0.1159 0.1083 | 0.1544 0.1483 | 0.5888  1.6920 |
| CIEE-1  CIEE-2 | 0.2716  0.2208 | 0.1906 0.1291 | 0.1367 0.0807 | **0.1314** **0.0671** | 0.5336  1.0352 |
| CIEF-1  CIEF-2 | 0.2565  0.2115 | 0.1791 **0.0877** | **0.0807** 0.1169 | 0.1809 0.1343 | 0.5804  1.0637 |
| CIEK-1  CIEK-2 | 0.2669  0.3833 | 0.1801 0.2194 | 0.1558 **0.1565** | **0.1391** 0.2398 | 0.5629  0.3858 |
| CIET-1  CIET-2 | 0.1328  0.1042 | 0.1070 **0.0708** | **0.0727** 0.1033 | 0.1114 0.1253 | 0.9339  1.1516 |

The bold numbers in the table indicate the minimum mean square error.
